# Supplementary material for: Synthesis of Sugar and Nucleoside Analogs and Evaluation of Their Anticancer and Analgesic Potentials
Source: Molecules. 2022 May 29;27(11):3499. doi: 10.3390/molecules27113499 (PMC9182362; doi:10.3390/molecules27113499)
Supplement: Supplementary file 1 [file molecules-27-03499-s001.zip › molecules-1728868-supplementary.pdf]

Supporting Information

Synthesis of Sugar and Nucleoside analogues and Evaluation of their Anticancer and Analgesic Potentials

Fahad Hussain[a], Fahad Imtiaz Rahman[a], Poushali Saha[a], Atsushi Mikami[b], Takashi Osawa[b], Satoshi Obika[b], S. M. Abdur Rahman[a,*]

[a] Department of Clinical Pharmacy and Pharmacology, Faculty of Pharmacy, University of Dhaka, Dhaka-1000, Bangladesh

[b] Graduate School of Pharmaceutical Sciences, Osaka University, 1-6 Yamadaoka, Suita, Osaka 565-0871, Japan.

Corresponding Author:

Professor Dr. S. M. Abdur Rahman

Department of Clinical Pharmacy and Pharmacology, Faculty of Pharmacy, University of Dhaka

Phone: +88(02)9661920-73, Ext. 8166 (office),

+8801732477343 (mobile);

Email: smarahman@du.ac.bd

1. Preparation of 1,2:5,6-Di-O-isopropylidene-α-D-allofuranose (2)

Starting compound **1** (7.5 g, 41.62 mmol) was stirred in room temperature for 24 hours with dry acetone (350 ml) and sulfuric acid (1.88 ml, 35.38 mmol) in the presence of copper sulfate (0.66 g, 4.16 mmol) to produce **1a** (Yield: 7.8g, 72%). Then the diisopropylidene **1a** (7g, 26.9 mmol) was dissolved in dichloromethane (50 ml) and 2,2,6,6-Tetramethylpiperidin-1-yl)oxyl (TEMPO) (0.126 g, 8.07 mmol) and KBr (0.32 g, 2.69 mmol) were added and the reaction mixture was stirred at room temperature for 2-5 minutes. After that 5% sodium hypochlorite (60 ml, 40.27 mmol) was poured dropwise to the reaction mixture for 1 hour at 0°C to produce α-D-ribo-hexofuranos-3-ulose **1b** (Yield: 4.7g, 67%). Compound **1b** (4.7 g, 18 mmol) was then dissolved in methanol (100 ml) and $NaBH_4$ was added at 0ºC temperature followed by vigorous stirring at room temperature for 2 hours to produce a clear viscous oil to white solid residue of compound **2** (Scheme S1). All analytical data were identical to those previously reported.

Yield: 4.12 g (59%), ^{1}H-NMR (301 MHz, $CDCl_3$) δ_H (ppm) = 5.82 (d, *J* = 3.8 Hz, 1H), 4.62 (t, *J* = 4.5 Hz, 1H), 4.33 (dd, *J* = 6.5, 4.8 Hz, 1H), 4.11-3.99 (m, 3H), 3.82 (dd, *J* = 8.6, 4.8 Hz, 1H), 2.54 (d, *J* = 8.3 Hz, 1H), 1.58 (s, 3H), 1.47 (s, 3H), 1.38 (s, 6H).

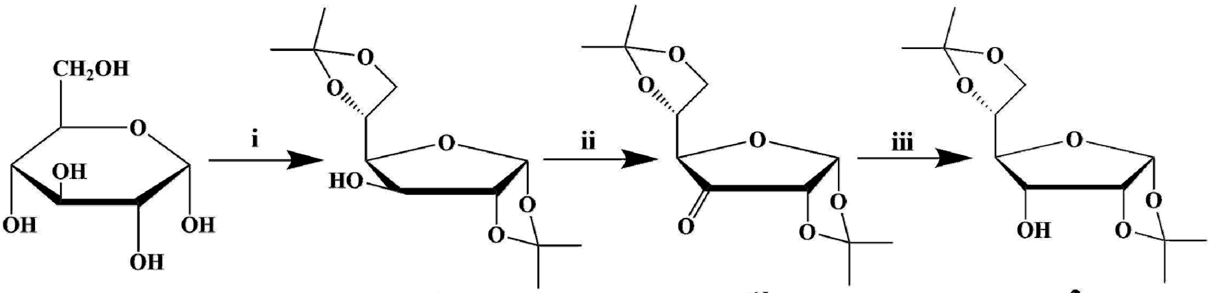

1 1a 1b 2

Scheme S1: Synthesis of compound **2**. Reagents and conditions: i) H_2SO_4, $CuSO_4$, $(CH_3)_2CO$, 72%; ii) TEMPO, NaOCl, CH_2Cl_2, KBr, 67%; iii) $NaBH_4$, CH_3OH, 59%.

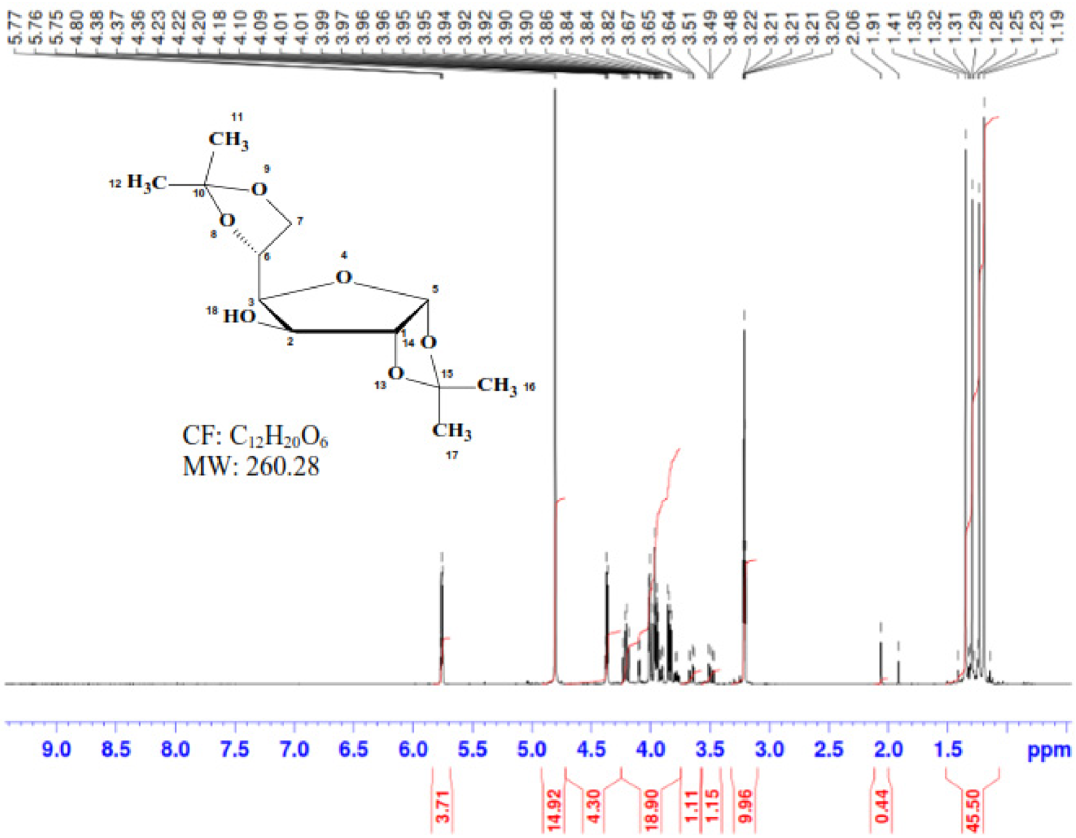

Figure S1: ^{1}H NMR spectrum of compound **1a**

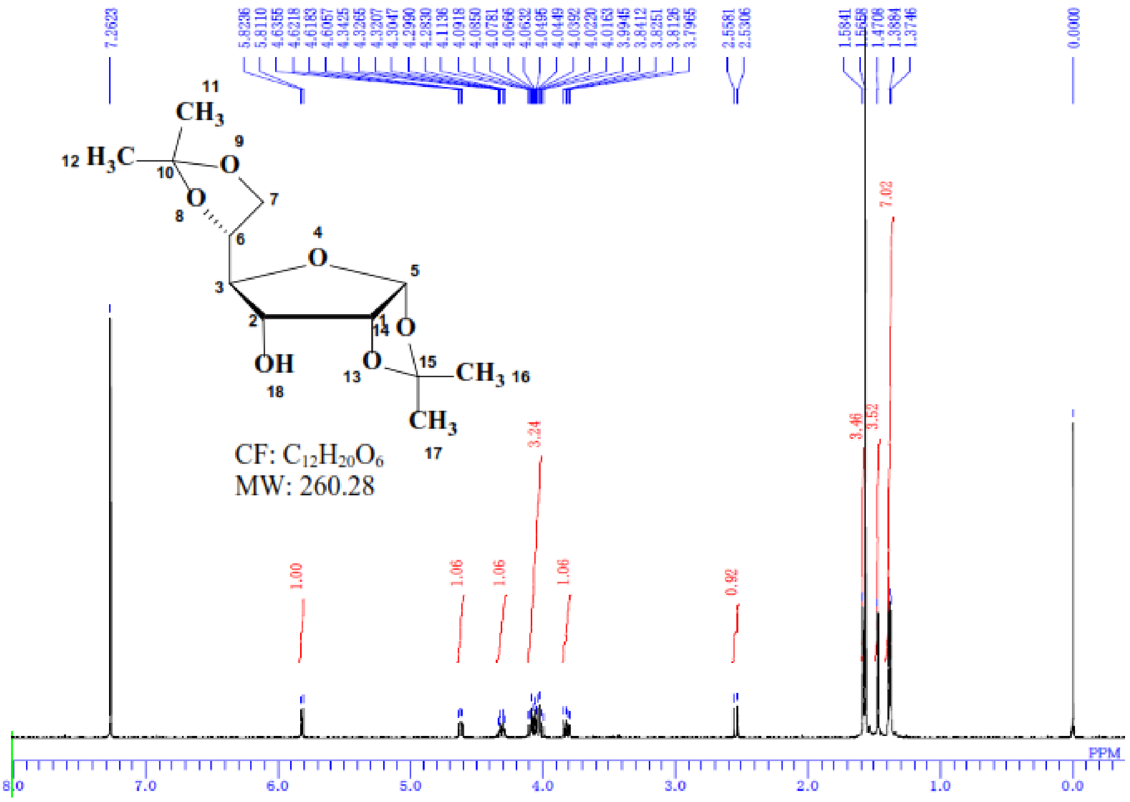

Figure S2: ^{1}H NMR spectrum of compound **2**

2. Preparation of 3-O-benzyl-4-C-hydroxymethyl-1,2-O-isopropylidene-α-D-ribofuranose (3)

Synthesis of compound **3** from compound **2** was done in four steps. Compound **2** (2.6 g, 10 mmol) was dissolved in dehydrated THF (30 ml) and NaH (0.5 g, 20.8 mmol) was added to the mixture and stirred for 1.5 hours at room temperature. Benzyl bromide (1.4 ml, 11.78 mmol) was added at 0°C and reaction was stirred for 24 hours to synthesize compound **2a** (Yield: 3.07g, 87%). Compound **2a** (2.9 g, 8.27 mmol) was then treated with 80% acetic acid (15 ml) and was stirred at room temperature to produce **2b** (Yield: 2.4g, 93%). Compound **2b** (2.4 g, 7.73 mmol) was then dissolved in an equal volume mixture of THF (4 ml) and distilled water (4 ml). Sodium periodate (1.6 g, 7.48 mmol) was then added in small portions over a 30-minute period followed by vigorous stirring for 3 hours to produce **2c** (Yield: 1.76 g, 82%). Finally, compound **2c** (1.75 g, 6.29 mmol) was dissolved in 1,4-dioxane (4.4 ml, 51.3 mmol) followed by the addition of 37% methanal (1.5 ml). NaOH (3.3 ml, 4M) was then added dropwise in the mixture over a 30-minute period and the reaction was stirred for 24 hours to produce compound **3** (Scheme 2)**.**

Yield: 1.37 g (53%), ^{1}H-NMR (500 MHz, $CDCl_3$) δ_H (ppm) = 7.34 (m, 5H), 5.77 (d, J = 2.4 Hz, 1H), 4.81 (d, J = 7.2 Hz, 1H), 4.65 (t, J = 1.9 Hz, 1H), 4.56 (d, J = 6.9 Hz, 1H), 4.22 (d, J = 3 Hz, 1H), 3.91 (d, J = 4.5 Hz, 2H), 3.79 (dd, J = 2.4, 7.2 Hz, 1H), 3.57 (t, J = 4.3 Hz, 1H), 2.35 (t, J = 4.2 Hz, 1H), 1.80 (dd, J = 2.1, 6.0 Hz, 1H), 1.63 (s, 3H), 1.56 (s, 3H).

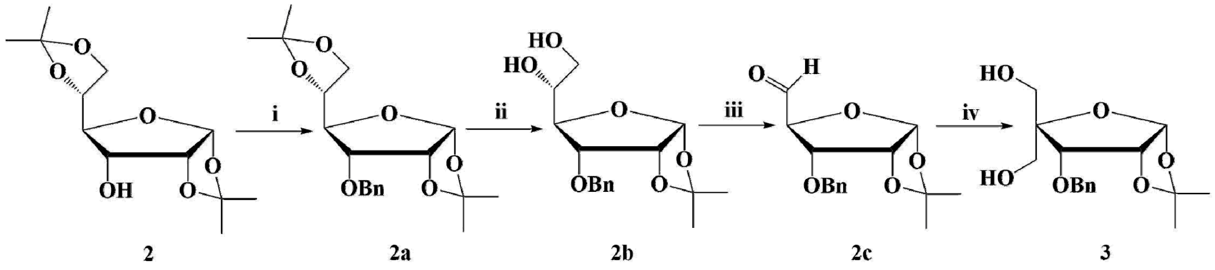

Scheme S2: Synthesis of compound **3**. Reagents and conditions: i) BnBr, NaH, THF, 87%; ii) 80% CH_3COOH, 93%; iii) $NaIO_4$, THF/H_2O (1:1), 82%; iv) 37% HCHO, NaOH, Dioxane, 54%.

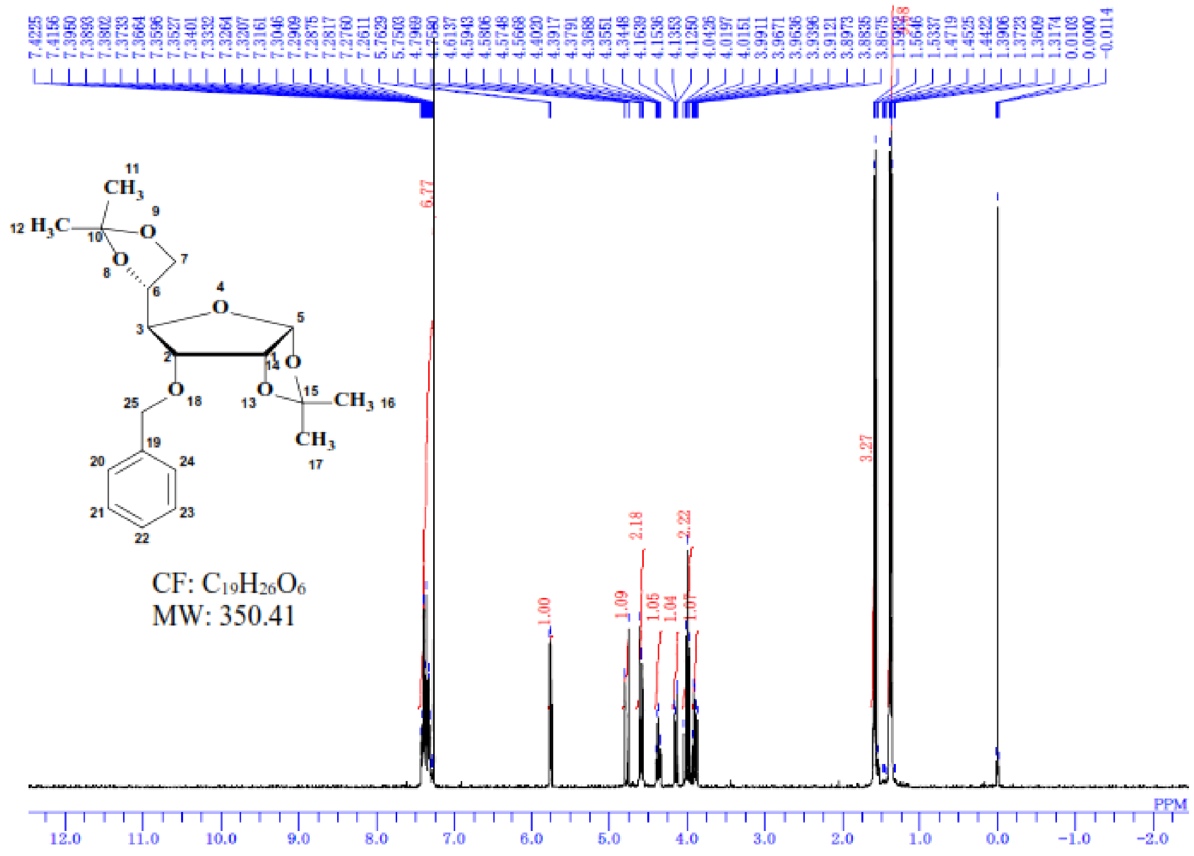

Figure S3: ^{1}H NMR spectrum of compound **2a**

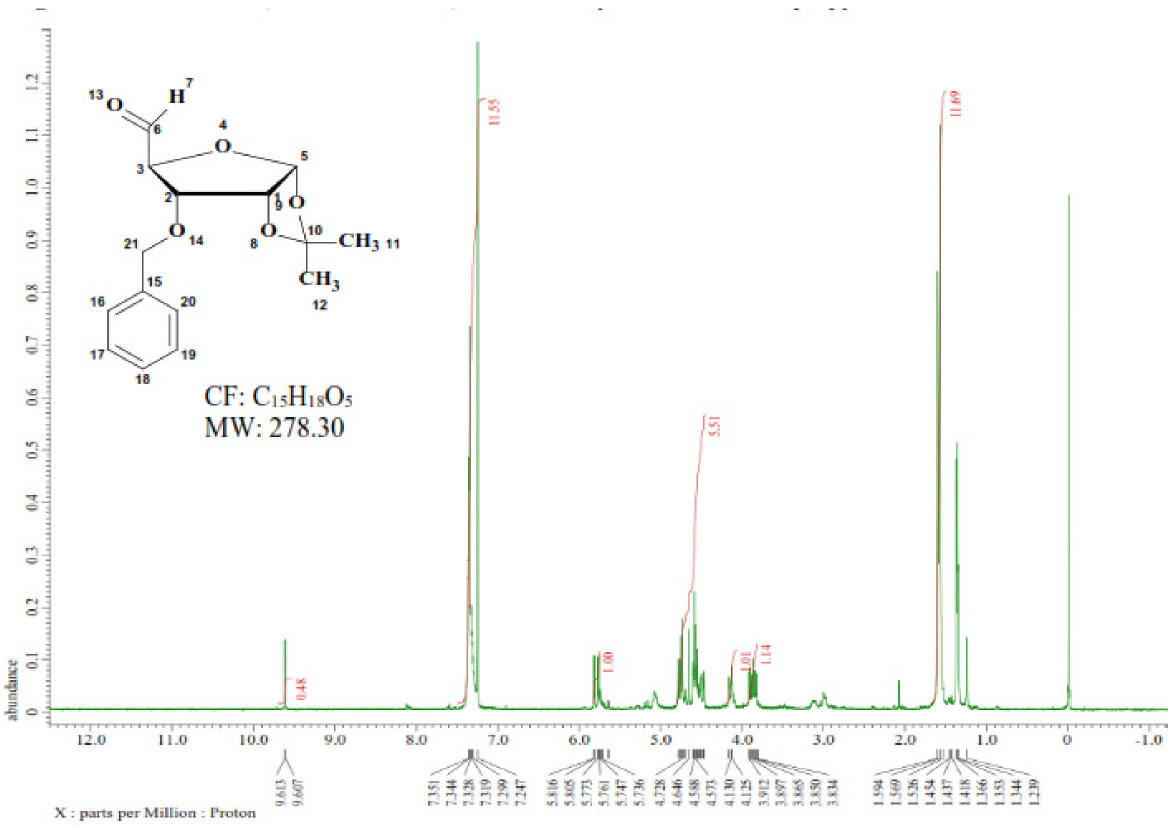

Figure S4: ^{1}H NMR spectrum of compound **2c**

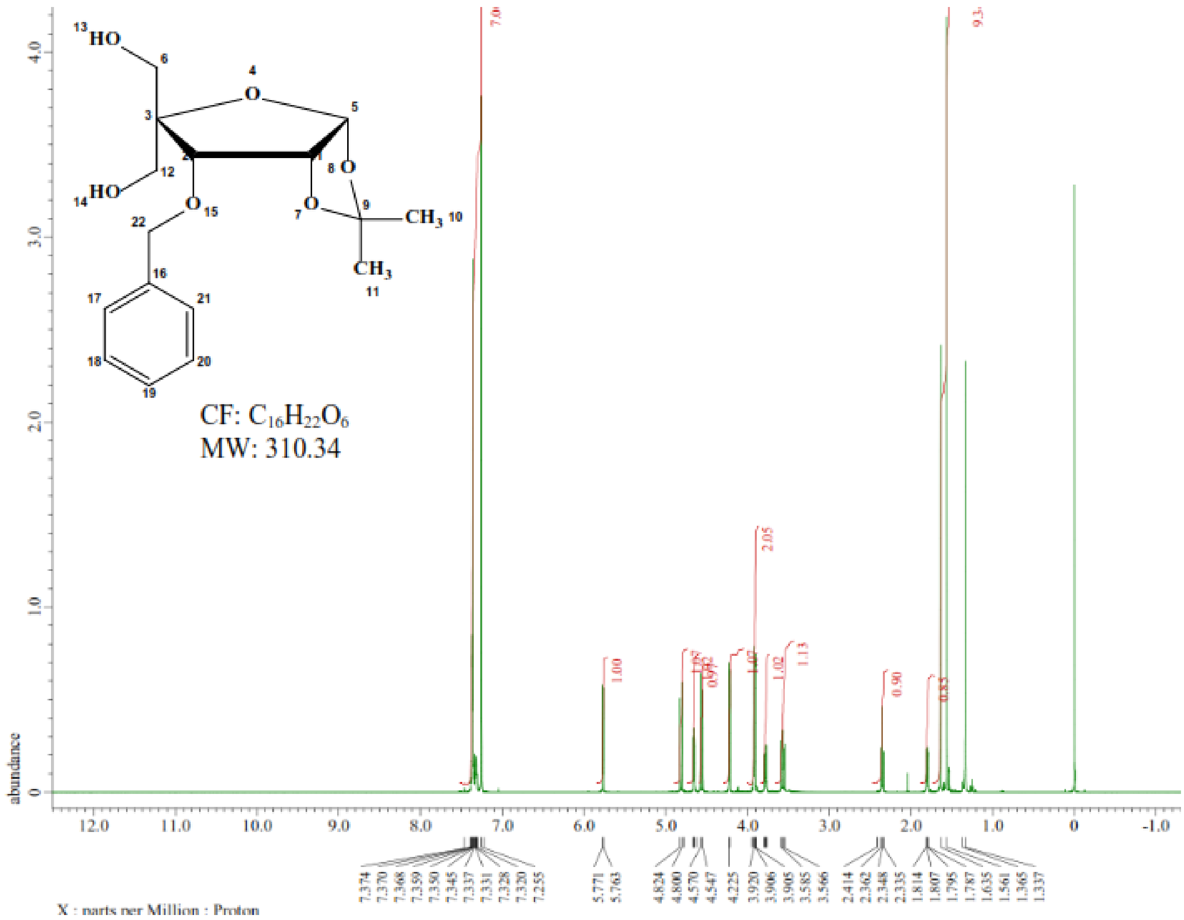

Figure S5: ^{1}H NMR spectrum of compound **3**

3. Preparation of 1-(4-C-acetoxymethyl-2-O-acetyl-3,5-di-O-benzyl-β-D-ribofuranosyl)thymine (4) and 1-(4-C-hydroxymethyl-2-hydroxyl-3,5-di-O-benzyl-β-D-ribofuranosyl)thymine (5)

Synthesis of nucleoside analogue **4** began from compound **3** in three steps. NaH (0.77 g, 19.3 mmol) was dissolved in DMF (10 ml) and added dropwise over a period of 30 minutes to a solution of Compound **3** (5 g, 16.11 mmol) in DMF (40 ml). The reaction mixture was then stirred for 1.5 hours at room temperature and benzyl bromide (2.3 ml, 19.3 mmol) was added in small portions to the reaction medium and stirred for about 4 hours to produce **3a** (Yield: 4.89 g, 76%). Compound **3a** (3.84 g, 9.58 mmol) was then taken in a reaction vessel along with acetic acid (20 ml) and acetic anhydride (4 ml, 42.31 mmol) at 0°C. Sulfuric acid (20 µl, 0.36 mmol) was added to the reaction mixture and the stirred for 1 hours at room temperature to produce **3b** (Yields: 3.8 g, 81%). Compound **3b** (3.8 g, 7.8 mmol) was dissolved in acetonitrile (30 ml) and BSA (9.5 ml, 39 mmol) and thymine (1.08 g, 8.58 mmol) was added and refluxed at 90°C for 45 minutes. TMSOTf (1.2 ml, 4.20 mmol) was added and refluxed again for 2.5 hours to produce nucleoside **4** (Scheme S3)**.**

Yield: 3.34 g (63%), ^{1}H-NMR (301 MHz, $CDCl_3$) δ_H (ppm) = 7.96 (s, 1H), 7.40-7.26 (m, 11H), 6.22 (d, *J* = 5.5 Hz, 1H), 5.41 (t, *J* = 5.5 Hz, 1H), 4.60 (t, *J* = 11.2 Hz, 1H), 4.52-4.40 (m, 4H), 4.19-4.09 (m, 2H), 3.77 (d, *J* = 10.1 Hz, 1H), 3.51 (d, *J* = 10.1 Hz, 1H), 2.10 (s, 3H), 2.04 (s, 3H), 1.52 (s, 3H).

Compound **4** (2.24 g, 4.06 mmol) was dissolved in methanol (70 ml) followed by the addition of 28% aqueous NH_3 (10 ml) and vigorously stirred for 24 hours to produce the nucleoside **5** (Scheme S3)**.**

Yield: 1.6 g (84%), ^{1}H-NMR (301 MHz, CD_3OD) δ_H (ppm) = 7.9 (s, 1H), 7.61 (s, 1H), 7.6-7.27 (m, 10H), 6.03 (d, *J* = 4.4 Hz, 1H), 4.8 (s, 1H), 4.55 (s, 1H), 4.5 (d, *J* = 5.4, 2H), 4.37 (dd, 4.5, 5.7 Hz, 1H), 4.28 (d, *J* = 5.7 Hz, 1H), 4.07 (dd, *J* = 7.2, 14.4 Hz, 1H), 3.57 (d, *J* = 2.7 Hz, 2H), 3.53 (s, 2H), 2.01 (s, 1H), 1.43 (s, 3H), 1.21 (t, *J* = 6.9 Hz, 1H).

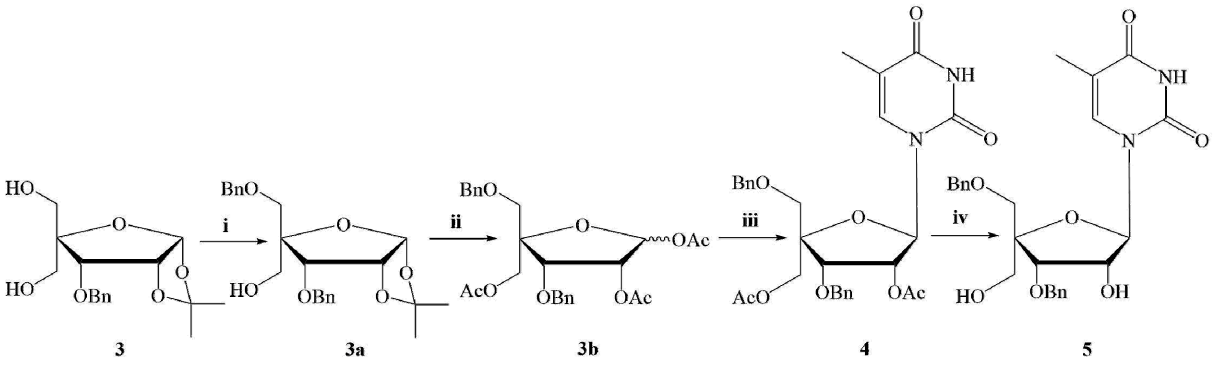

Scheme S3: Synthesis of compounds 4 and 5. Reagents and conditions: i) BnBr, NaH, DMF, 76%; ii) $(CH_3CO)_2O$, H_2SO_4, CH_3COOH, 81%; iii) Thymine, BSA, TMSOTf, CH_3CN, reflux at 90ºC, 63%; iv) 28% aq. NH_3, CH_3OH, 84%.

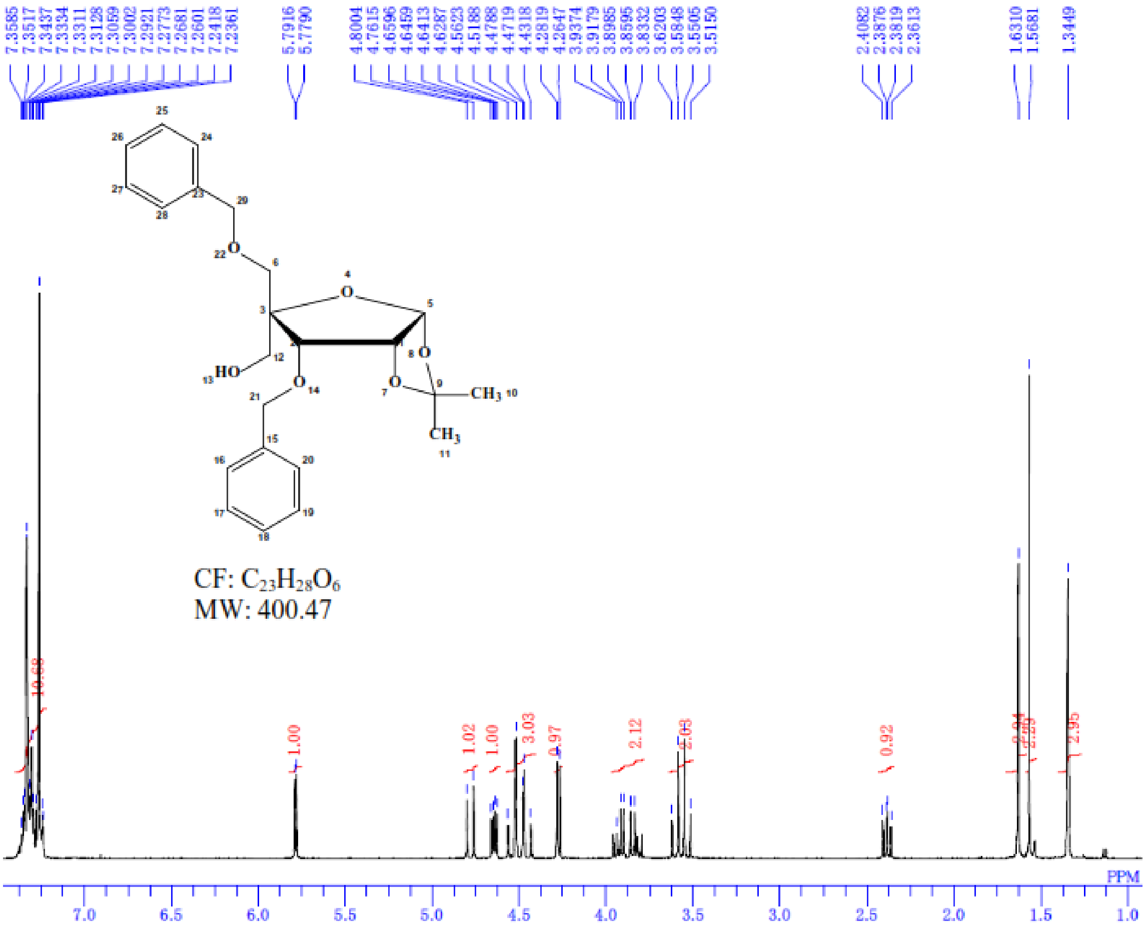

Figure S6: ^{1}H NMR spectrum of compound **3a**

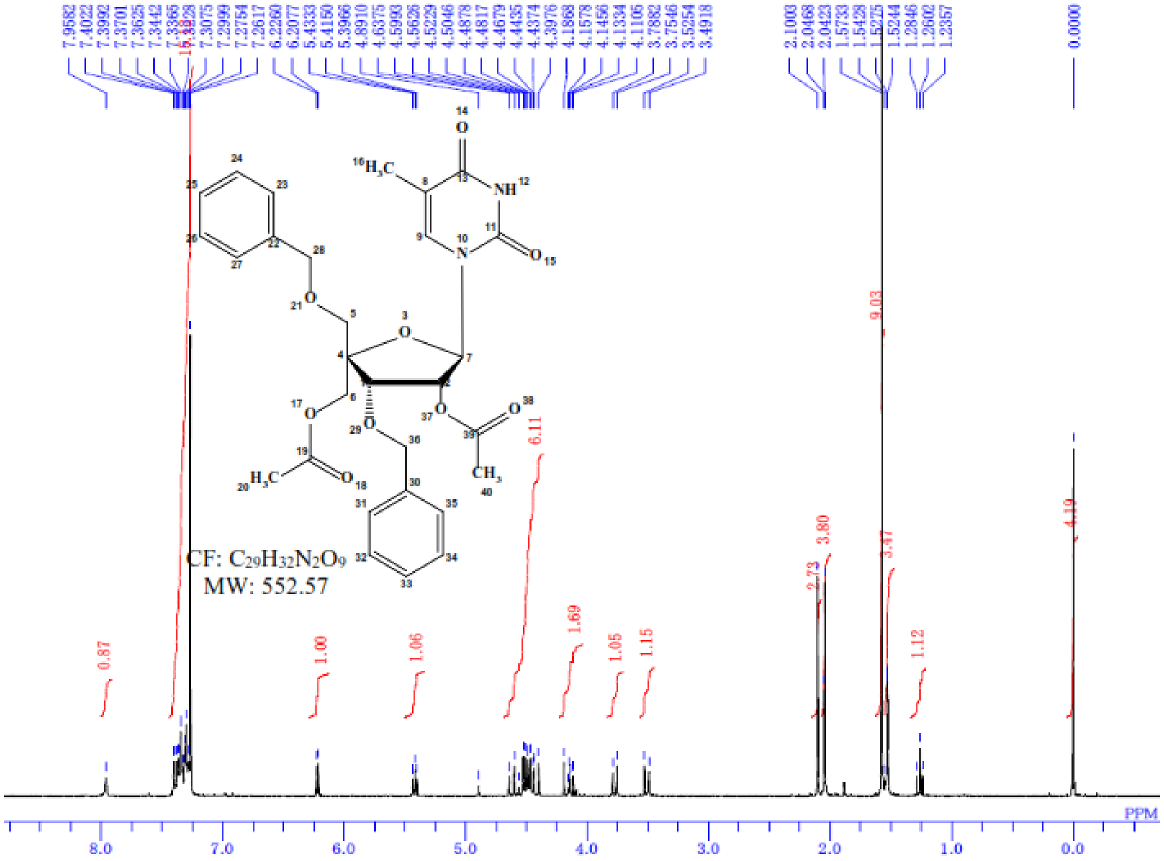

Figure S7: ^{1}H NMR spectrum of compound **4**

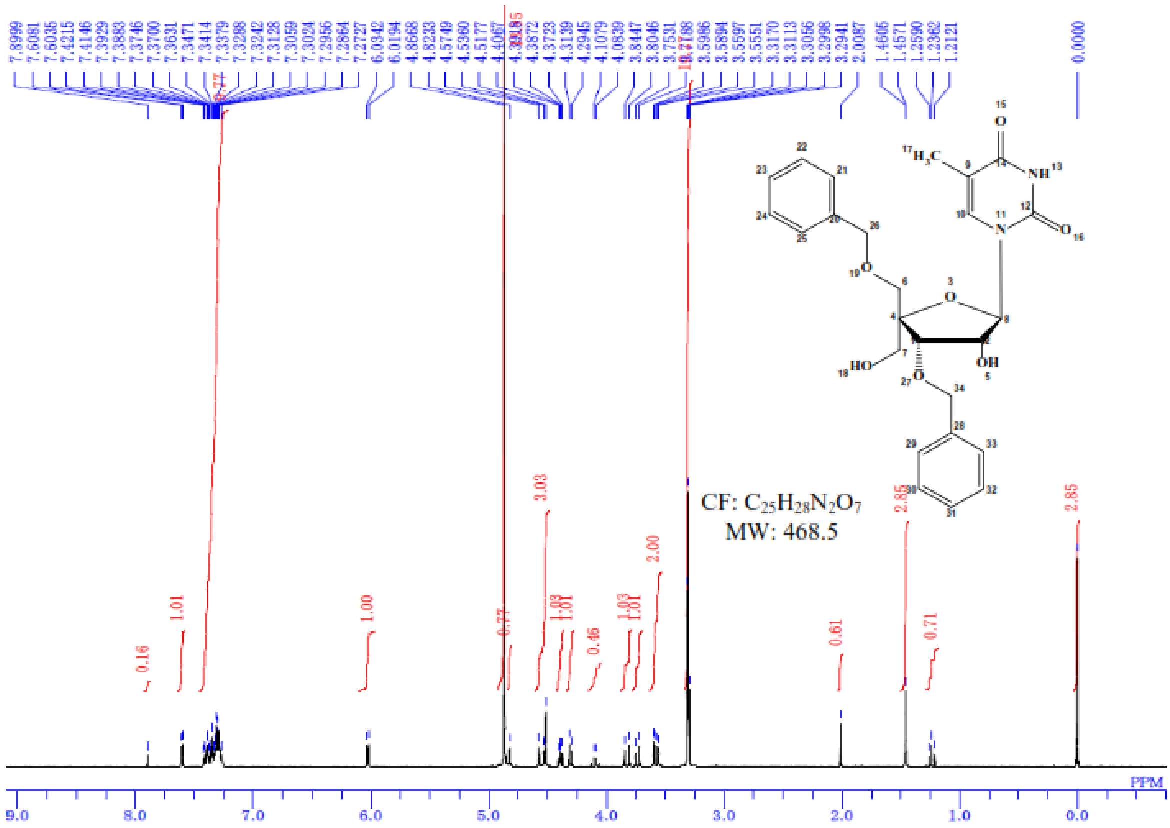

Figure S8: ^{1}H NMR spectrum of compound **5**

4. Preparation of 3’, 5’-di-O-benzyl-4’-C-tert-butyldiphenylsiloxymethyl-5-methyluridine (6)

Compound **6** was produced from **3** via four steps. First, compound **3a** was prepared from compound **3** according to Scheme S3. Compound **3a** (10 g, 25 mmol) was dissolved in DMF (100 ml) and imidazole (3.74 g, 54.9 mmol) and TBDPSCl (9.7 g, 37.4 mmol) were added in the reaction medium and stirred for 24 hours at room temperature to produce **3c** (Yield: 15.3 g, 96%). Compound **3c** (15.3 g, 23.95 mmol) was then dissolved in acetic acid (22.5 ml, 393.4 mmol) and acetic anhydride (26.2 ml, 277.8 mmol) was added to the reaction mixture. Concentrated sulfuric acid (0.1 ml, 1.9 mmol) was then added to the reaction mixture at 0°C and stirred for 3 hours more to produce **3d** (Yield: 12.8g, 78%). Finally compound **3d** (12.8 g, 18.74 mmol) was dissolved in acetonitrile (60 ml) and BSA (15.6 ml, 63.7 mmol) and thymine (2.38 g, 18.92 mmol) were added in the reaction mixture and refluxed at 90°C for 45 minutes. After that, TMSOTf (1.38 ml, 7.87 mmol) was added and refluxed again for 4 hours to produce nucleoside **6 (**Scheme 4)**.**

Yield: 9.3 g (53%), ^{1}H-NMR (301 MHz, $CDCl_3$) δ_H (ppm) = 7.97 (s, 1H), 7.67-7.60 (m, 5H), 7.47-7.21 (m, 16H), 5.95 (d, J = 4.8 Hz, 1H), 4.70 (dd, J = 11.0, 19.6 Hz, 2H), 4.50 (s, 2H), 4.39 (dd, J = 4.8, 11.1 Hz, 1H), 4.29 (d, J = 6.0 Hz, 1H), 3.76 (dd, J = 10.8, 28.5 Hz, 2H), 3.6 (dd, J = 8.7, 10.5 Hz, 2H), 3.5 (s, 1H), 1.59 (s, 3H), 1.06 (s, 9H).

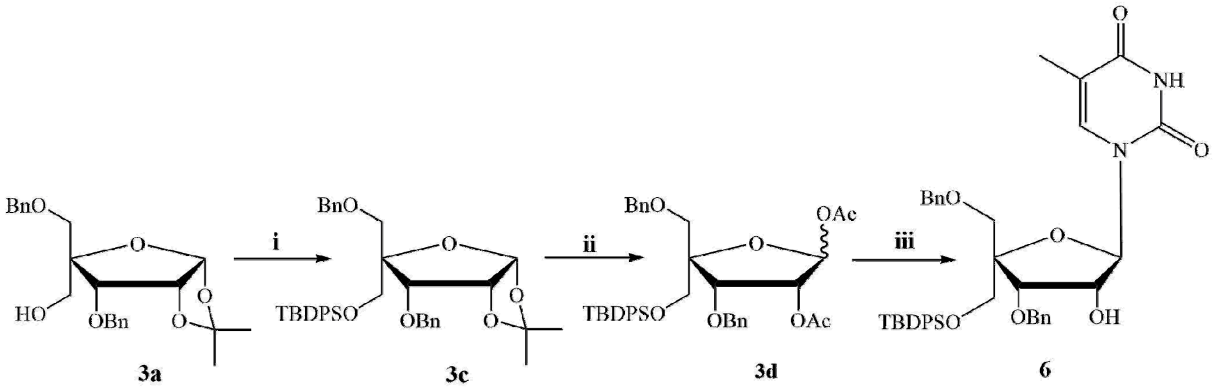

Scheme S4: Synthesis of compound 6. Reagents and conditions: i) TBDPSCl, Imidazole, DMF, 96%; ii) $(CH_3CO)_2O$, H_2SO_4, CH_3COOH, 78%; iii) Thymine, BSA, TMSOTf, CH_3CN, reflux at 90°C, 53%.

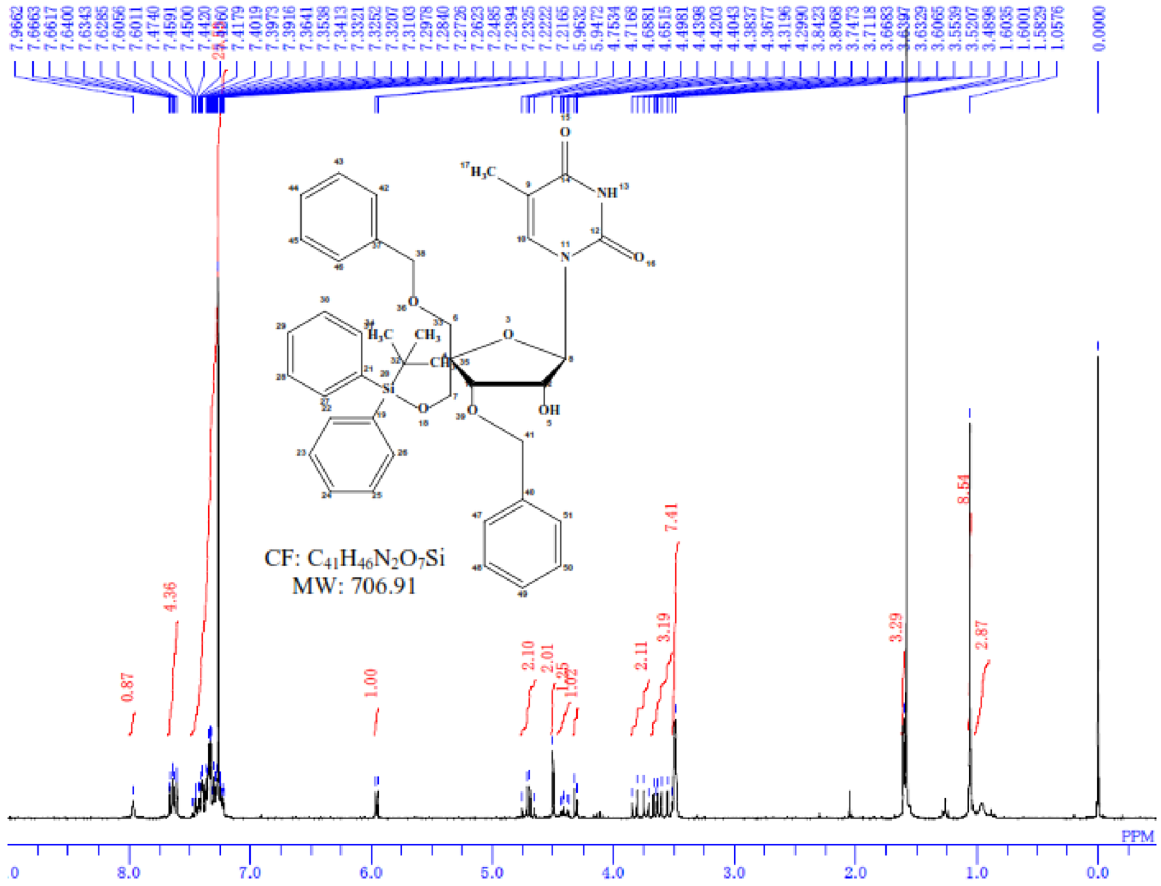

Figure S9: ^{1}H NMR spectrum of compound **6**

5. Preparation of (1S,3R,4R,7S)-7-hydroxy-1-hydroxymethyl-3-(thymin-1-yl)-2,5-dioxabicyclo [2.2.1]heptane (7) and ((1R,3R,4R,7S)-7-hydroxy-3-(5-methyl-2,4-dioxo-3,4-dihydropyrimidin-1(2H)-yl)-2,5-dioxabicyclo[2.2.1]heptan-1-yl)methyl isobutyrate (8)

Locked nucleoside **7** was produced from compound **3** in six steps according to previously reported methods with slight modifications. First, compound **3a** was prepared from compound **3** according to Scheme S3. Compound **3a** (5 g, 12.48 mmol) was dissolved in dichloromethane (25 ml) and triethylamine (12 ml, 86.45 mmol), DMAP (0.23 g, 1.87 mmol) and TsCl (3.81 g, 20 mmol) were added to the mixture and stirred for 24 hours to produce **3e** (Yield: 6.7 g, 96%). Compound **3e** (6.9 g, 12.44 mmol) was dissolved in acetic acid (25 ml, 435.4 mmol) in a reaction vessel and acetic anhydride (5.87 ml, 62.2 mmol) was added to the mixture. Concentrated sulfuric acid (0.12 ml, 2.28 mmol) was added at 0°C and stirred for 3 hours to produce compound **3f** (Yield: 5.8 g, 78%). Compound **3f** (9.91 g, 16.55 mmol) was dissolved in acetonitrile (50 ml) and BSA (10.2 ml, 41.7 mmol) and thymine (3.17 g, 25.16 mmol) were added in the reaction mixture and refluxed for 1 hour at 85°C. After cooling the reaction mixture, TMSOTf (1.38 ml, 7.87 mmol) was added dropwise and refluxed again at 85°C for 4 hours to produce **3g** (Yield: 8.1 g, 74%). Compound **3g** (5.58 g, 8.39 mmol) was dissolved in tetrahydrofuran (28 ml) and methanol (70 ml, 1.73 mmol) was added followed by potassium bicarbonate (4.06 g, 29.38 mmol) to produce bridged nucleoside **3h** (Yield: 3.39 g, 90%). Finally, compound **3h** (2.7 g, 6 mmol) was dissolved in ethanol (42 ml) and 20% palladium hydroxide on carbon (1.39 g, 9.9 mmol) was added and the mixture was stirred for 4 hours to produce bridged nucleoside **7** (Scheme S5).

Yield: 1.57 g (97%), ^{1}H-NMR (301 MHz, DMSO-D$_6$) δ_H (ppm) = 11.33 (s, 1H), 7.61 (s, 1H), 5.63 (d, *J* = 4.5 Hz, 1H), 5.4 (s, 1H), 5.17 (t, *J* = 5.7 Hz, 1H), 4.1 (s, 1H), 3.9 (d, *J* = 4.1 Hz, 1H), 3.81 (d, *J* = 7.6 Hz, 1H), 3.75 (d, *J* = 5.5 Hz, 2H), 3.62 (d, *J* = 7.6 Hz, 1H), 1.77 (s, 3H).

The new bridged nucleoside analogue **8** was synthesized by reacting the compound **7** (0.6 g, 2.22 mmol) with isobutyric anhydride (0.55 ml, 3.31 mmol) in pyridine (23 ml, 285.53 mmol) for 24 hours. The concentrated crude was purified by column chromatography (9.5:0.5 (v/v) $CHCl_3/CH_3OH$) to produce solid crystalline compound **8** (Scheme S5).

Yield: 0.42g (56%), R_f = 0.7 (9:1 (v/v) $CHCl_3/CH_3OH$.); ^{1}H NMR (400 MHz, $CDCl_3$) δ_H (ppm) = 8.30 (s, 1H), 7.39 (s, 1H), 5.61 (s, 1H), 4.63 (d, *J* = 12.8 Hz, 1H), 4.52 (s, 1H), 4.35 (d, *J* = 12.8 Hz, 1H), 4.08 (d, *J* = 8.3 Hz, 1H), 3.90 (d, *J* = 8.3 Hz, 2H), 2.85 (d, *J* = 5.0 Hz, 1H), 2.68 (m, 1H),

1.94 (s, 3H), 1.25 (m, 6H). ^{13}C NMR (126 MHz, CD_3OD) δ: 177.83, 166.36, 151.76, 136.15, 110.87, 88.55, 87.91, 80.81, 72.50, 71.16, 60.53, 35.13, 19.39, 12.68. HRMS calc. for $C_{15}H_{20}N_2O_7Na$ $[M+Na]^+$: 363.1160; found: 363.1163.

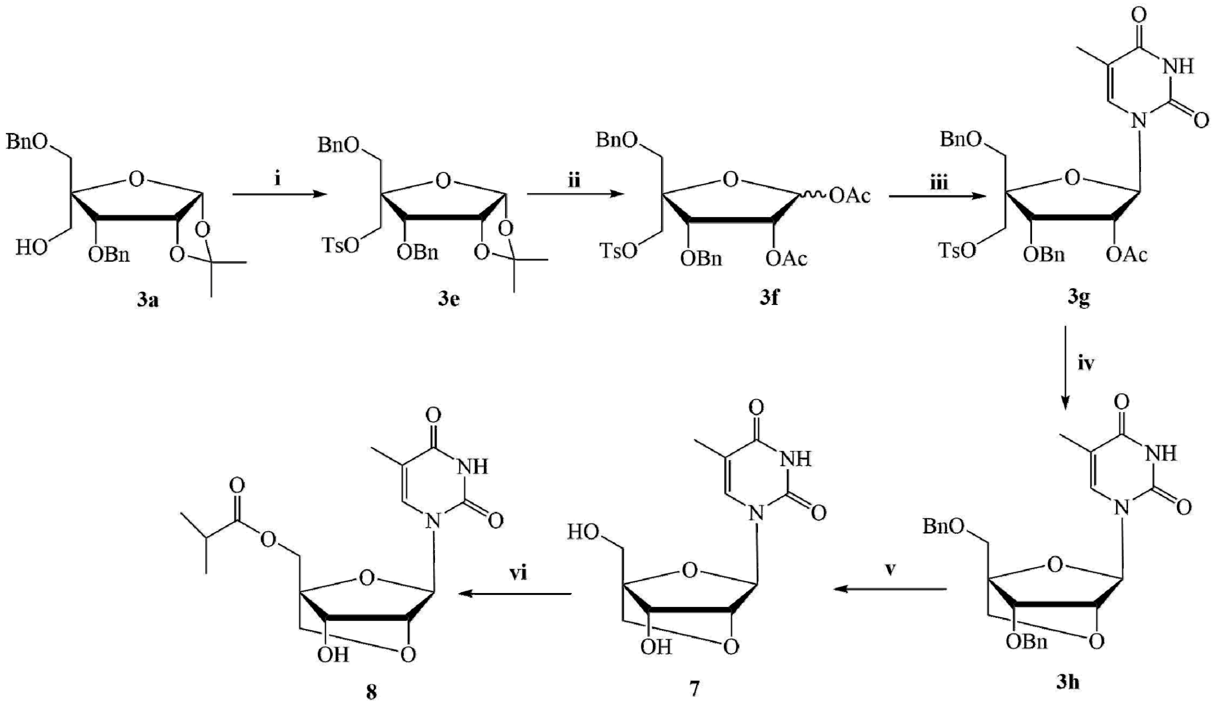

Scheme S5: Synthesis of compounds **7** and **8**. Reagents and conditions: i) TsCl, DMAP, $N(C_2H_5)_3$, CH_2Cl_2, 0°C > rt, 96%; ii) $(CH_3CO)_2O$, H_2SO_4, CH_3COOH, 78%; iii) Thymine, BSA, TMSOTf, CH_3CN, reflux at 90°C, 74%; iv) K_2CO_3, THF, CH_3OH, 90%; v) 20% $Pd(OH)_2$, over carbon, C_2H_5OH, 97%; vi) Isobutyric anhydride, pyridine, 56%.

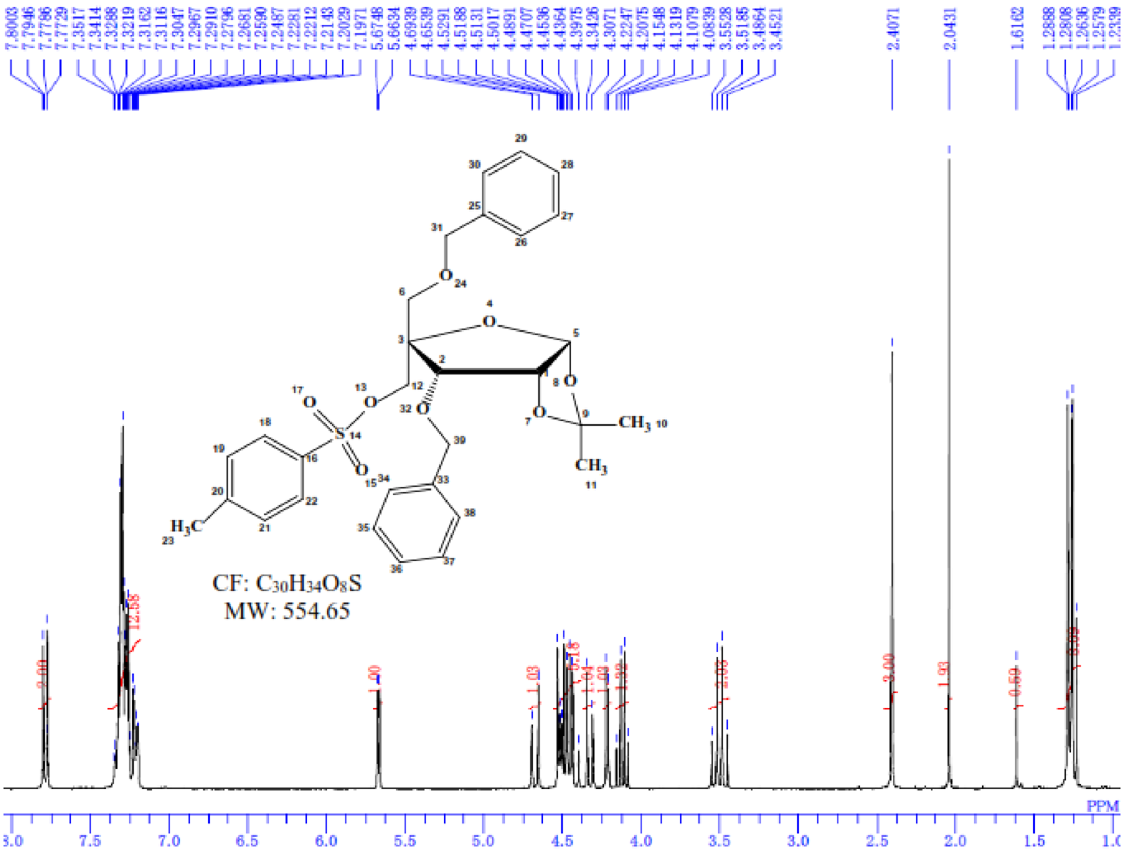

Figure S10: ^{1}H NMR spectrum of compound **3e**

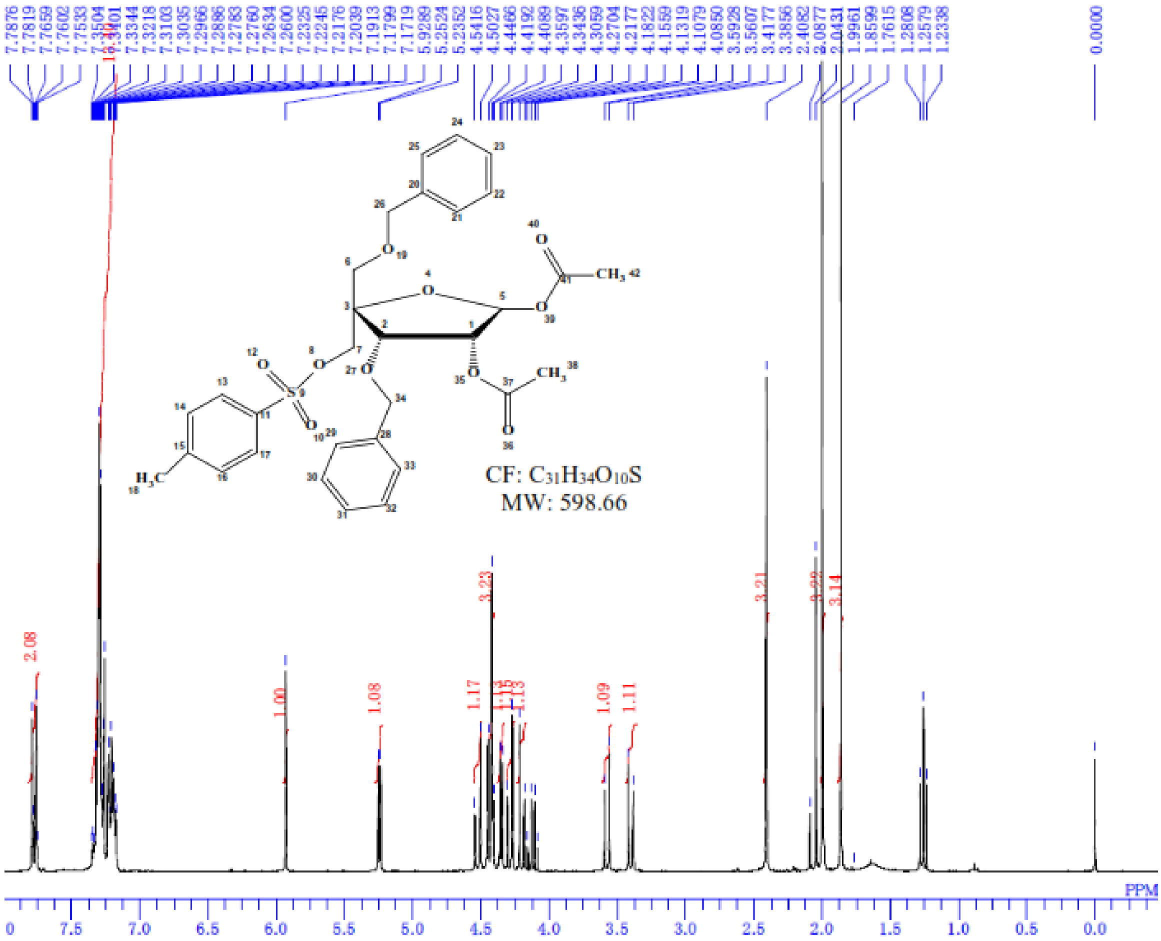

Figure S11: ^{1}H NMR spectrum of compound **3f**

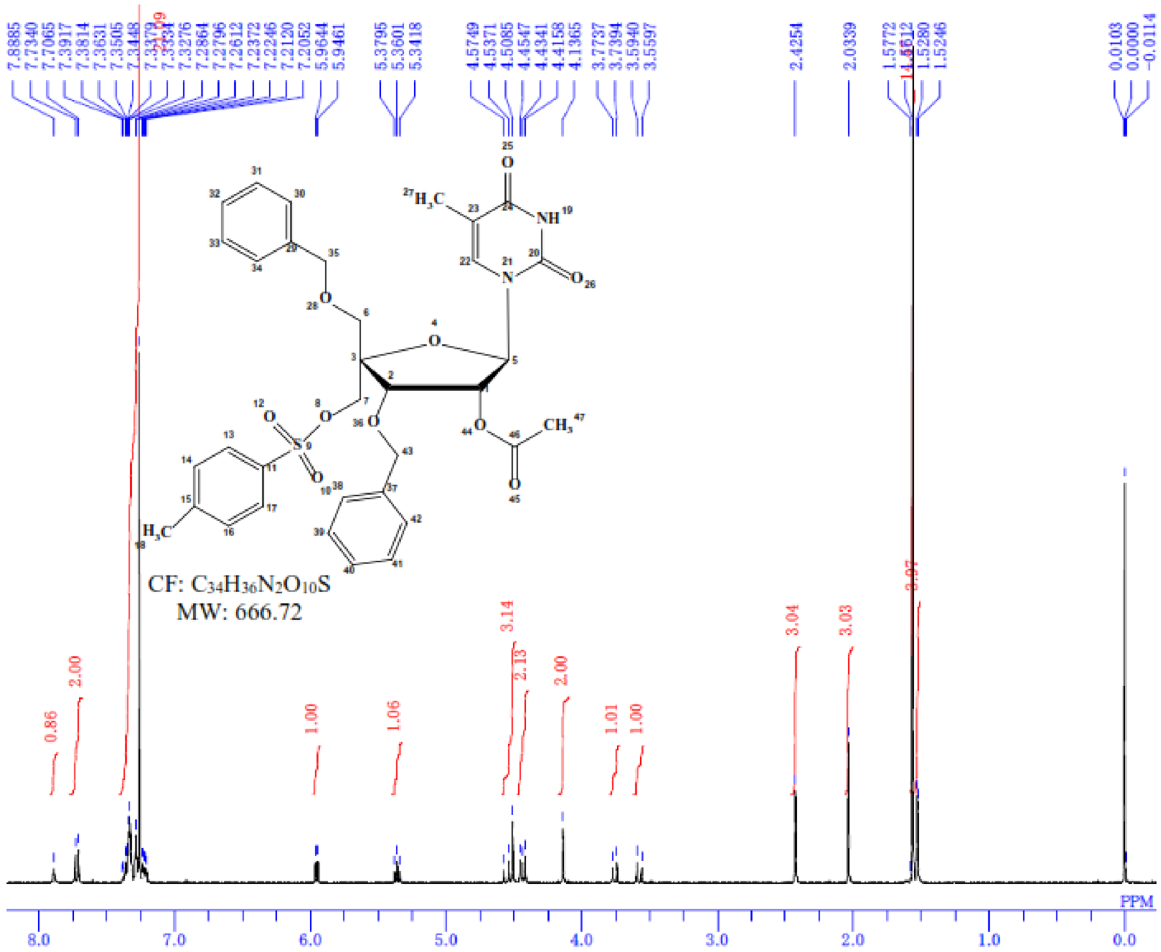

Figure S12: ^{1}H NMR spectrum of compound **3g**

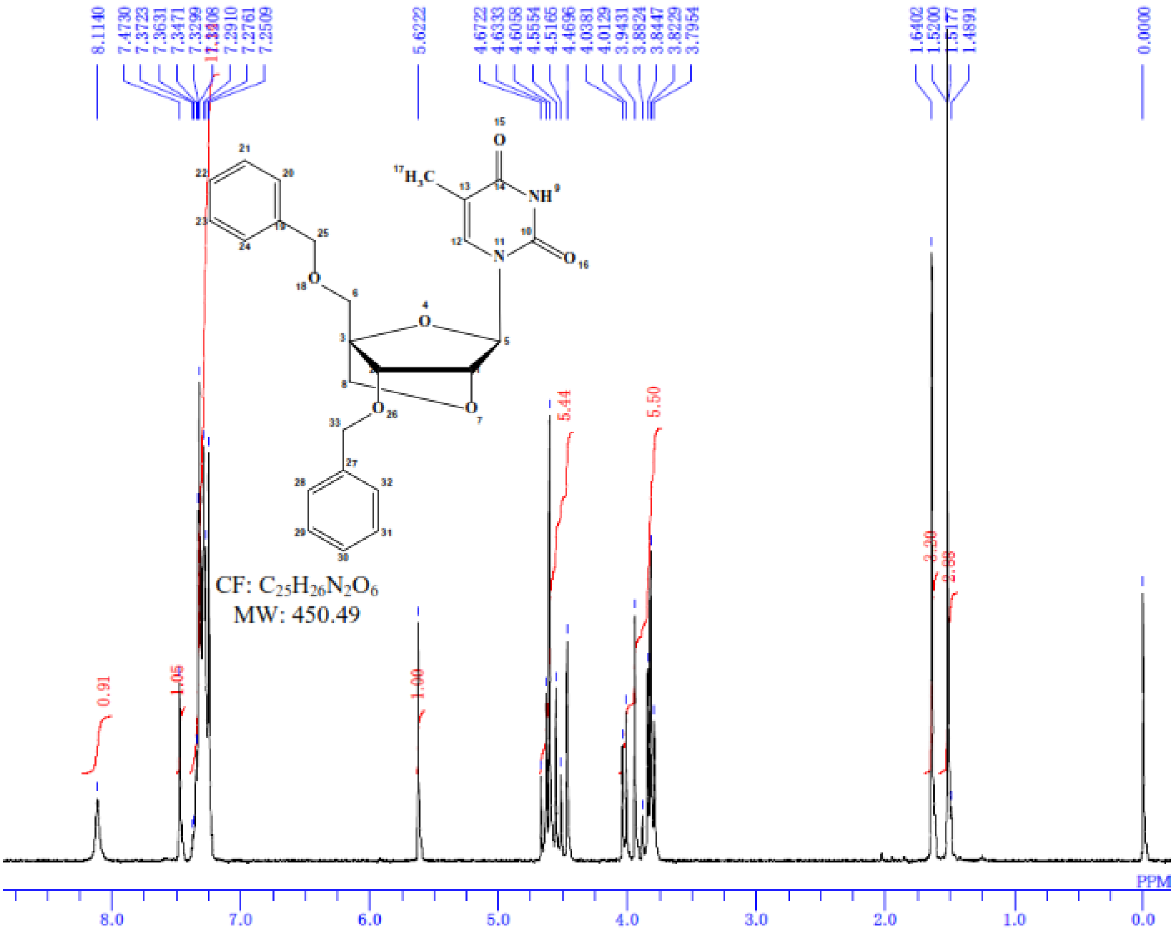

Figure S13: ^{1}H NMR spectrum of compound **3h**

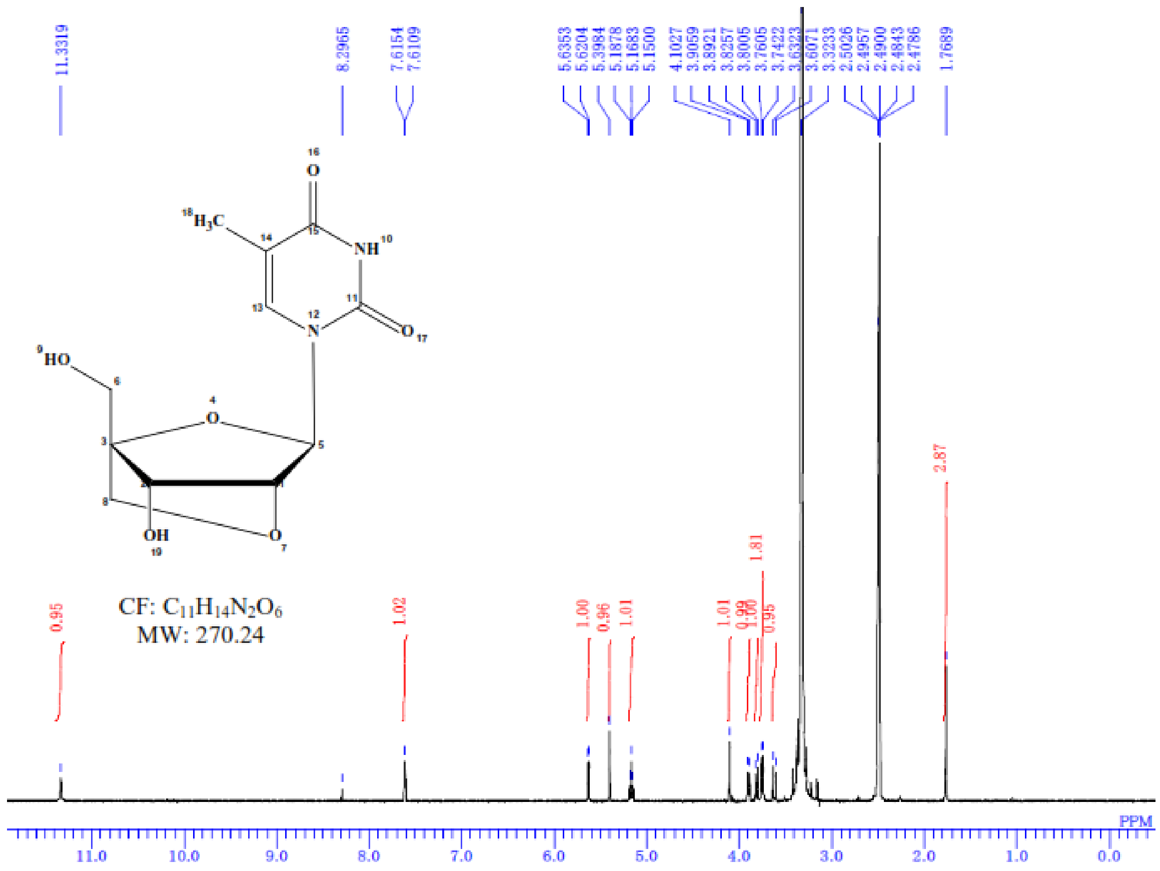

Figure S14: ^{1}H NMR spectrum of compound **7**

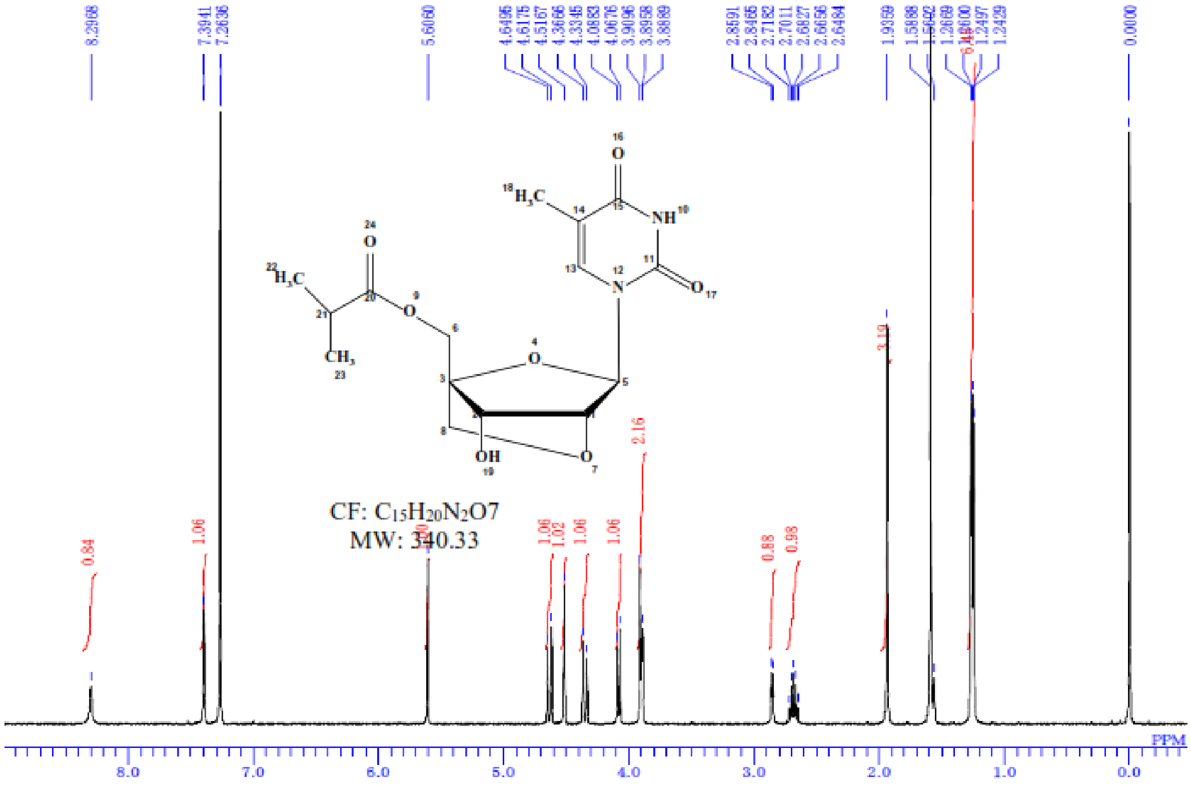

Figure S15: ^{1}H NMR spectrum of compound **8**

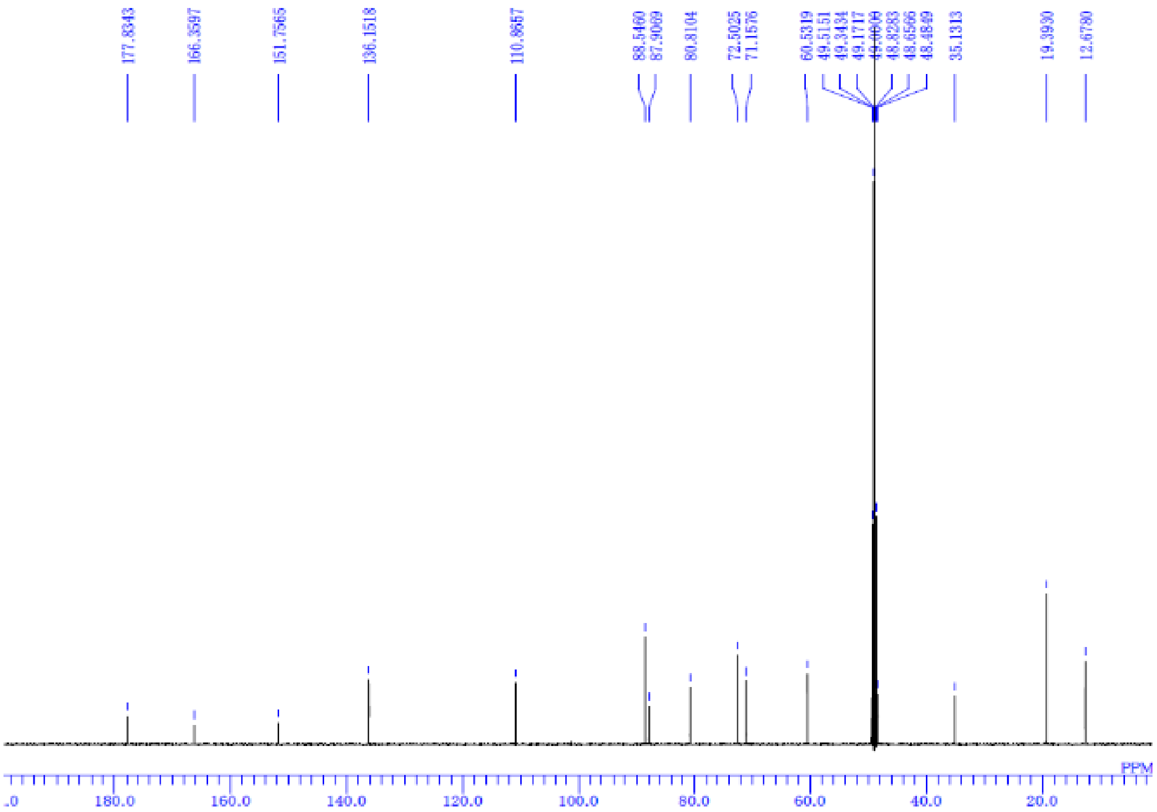

Figure S16: [1]C NMR spectrum of compound **8**

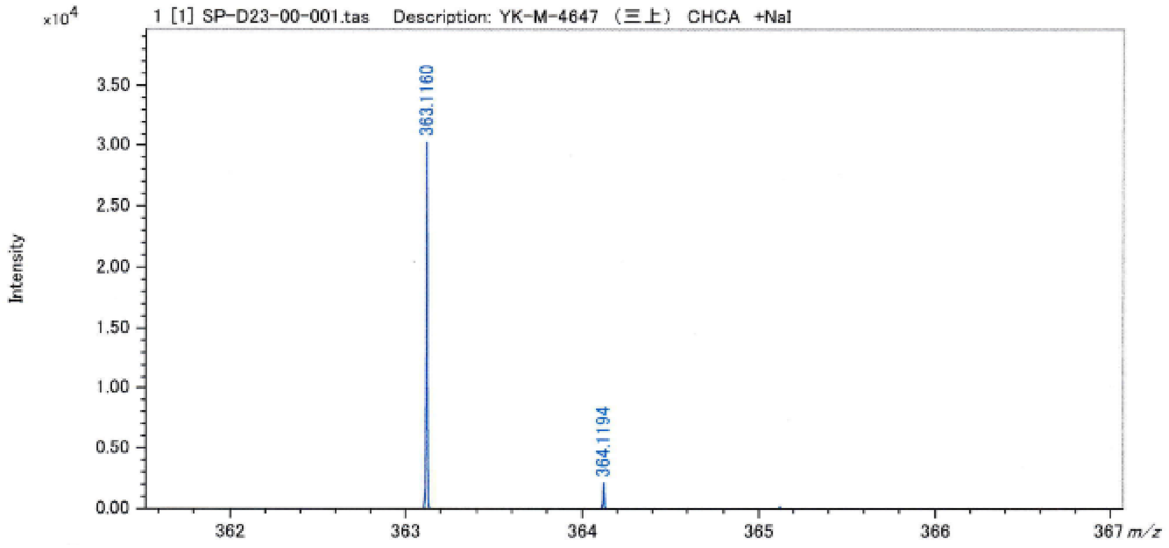

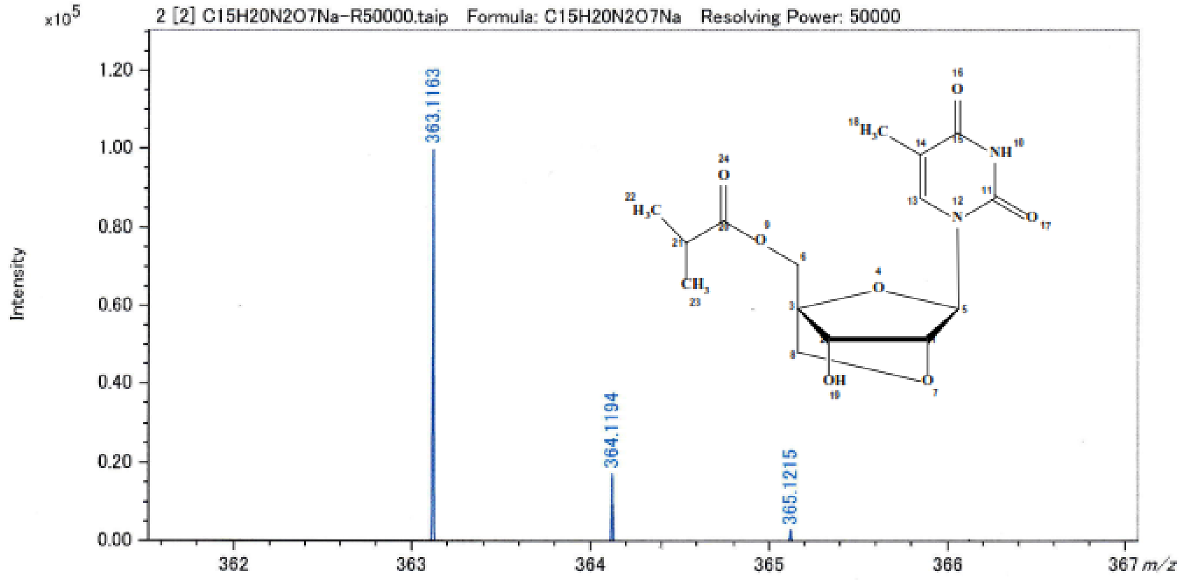

Elemental Composition Estimation

Parameters:

Mass	Tolerance	Electron Mode	Charge	DBE Range	Max Results
363.11602 ± 0.00182	5.0 ppm	Odd/Even	+1	−0.5 − 200.0	100

Elements

C 0 − 15 H 0 − 20 N 0 − 2 O 0 − 7 Na 0 − 1

Results:

#	Formula	Mass	DBE	Abs. Error (u)	Error (u)	Error (ppm)
1	C15 H20 N2 O7 Na	363.11627	6.5	0.00025	−0.00025	−0.70

Figure S17: MALDI-TOF mass spectra of spectrum of compound **8**
